# Revealing SO_2_ and CO_2_ adsorption features on forsterite *via* IR spectroscopy and automated computational approaches†

**DOI:** 10.1039/d5cp01699a

**Published:** 2025-06-03

**Authors:** Eric Mates-Torres, Guillermo Escolano Casado, Lorenzo Mino, Nadia Balucani, Piero Ugliengo, Albert Rimola

**Affiliations:** a Departament de Química, Universitat Autònoma de Barcelona 08193 Bellaterra Catalonia Spain eric.mates@uab.cat albert.rimola@uab.cat; b Dipartimento di Chimica and Nanomaterials for Industry and Sustainability (NIS) Centre, Università degli Studi di Torino via P. Giuria 7 I-10125 Torino Italy lorenzo.mino@unito.it; c Dipartimento di Chimica, Biologia e Biotecnologie, Università degli Studi di Perugia Via Elce di Sotto 8 I-06123 Perugia Italy

## Abstract

The interaction between interstellar molecules and silicate dust plays a critical role in the chemical evolution of interstellar and circumstellar environments. In this work, we combine *in situ* infrared (IR) spectroscopy with automated density functional theory (DFT) calculations to investigate the adsorption and vibrational signatures of CO_2_ and SO_2_ on forsterite surfaces. Experimental IR spectra collected under cryogenic conditions reveal coverage- and temperature-dependent features that evolve from physisorbed to chemisorbed regimes. To interpret these observations, we construct theoretical spectra from a large ensemble of adsorption configurations across multiple surface terminations, weighted by their Boltzmann distributions at 100 K and by a per-surface abundance factor. The resulting spectra reproduce key experimental features, enabling the identification of binding trends. For CO_2_, we predict the transition from weakly bound species to carbonate-like modes at lower frequencies. For SO_2_, our simulations identify the dominant bands due to bidentate and tridentate chemisorption. This integrative approach highlights the importance of surface morphology and thermodynamic weighting in reconciling theory and experiments providing a framework for the spectroscopic analysis of molecular adsorption on interstellar dust analogs.

## Abstract 2

A pipeline combining automated calculations and *in situ* IR spectroscopy of CO_2_ and SO_2_ adsorption on forsterite reveals the structural origins of astrochemically relevant vibrational features *via* Boltzmann and Wulff-weighted spectra reconstruction.

## Graphical abstract

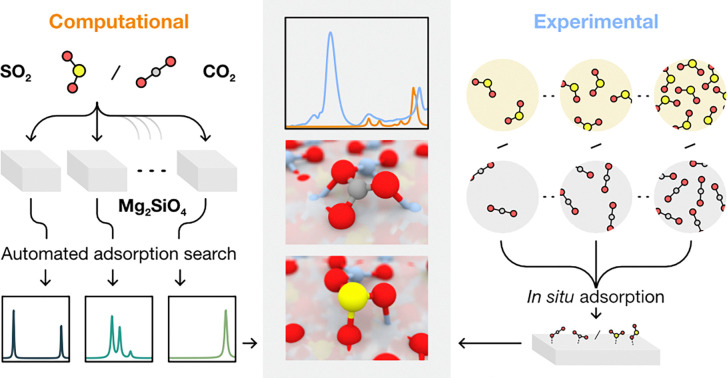

## Introduction

Silicates are ubiquitous in cosmic and early-Earth environments. This family of minerals, particularly forsterite (Mg_2_SiO_4_) and enstatite (MgSiO_3_), have been observed in protoplanetary disks, around young stellar objects (YSOs) and conforming dust shells around evolved stars, both in their crystalline and amorphous forms.^1–3^ While dust grains grown in the interstellar medium (ISM) are expected to contain purely amorphous silicates,^4^ the upper limit for the fraction of crystalline silicate grains injected into the ISM is up to 20%,^1^ yielding a total fraction of crystalline to amorphous mineral of 10% in active starburst regions.^5^ Silicates are also abundant in chondritic meteorite samples recovered on Earth,^6,7^ in interplanetary dust particles (IDPs) collected in the stratosphere^8,9^ and in the nuclei of the 81P/Wild (trapped within the aerogel of the Stardust mission)^10^ and 67P/CG comets.^11^ Thus, assessing the interactions between common interstellar molecules and forsterite is key to understand the primary steps of the ice growth on interstellar dust particles, to rationalize molecular abundance in diffuse and molecular clouds, and to decipher the formation of interstellar compounds, including those categorized as complex organic molecules (iCOMs). For instance, quantum chemical computations based on a cluster approach to model forsterite grain particles have shown that simple S-bearing species strongly bind on the silicate surfaces, potentially accounting for the missing sulfur in the gas phase in observational spectra.^12^ Calculations on crystalline forsterite surfaces have also elucidated how H_2_ and H_2_O may form from the adsorption, diffusion and reaction of two H atoms and H addition to O, respectively.^13,14^ Combined theoretical and synthetic laboratory experiments showcasing the reactivity of HCN and H_2_CO on forsterite surfaces have also shown promising results to rationalize the catalytic potential of this mineral for the formation of nucleobases and sugars, respectively.^15–18^

To realistically model the interactions between silicates and key interstellar molecules in a way that can be extrapolated to astronomical observations, quantum chemical calculations and spectroscopic laboratory measurements must work in tandem. In this regard, recent work by some of us has been devoted to understanding the true nature of the interactions between forsterite and HCN by fitting calculated infrared (IR) spectra to that obtained in low-temperature laboratory experiments, revealing a mainly dissociative mechanism undergoing on the forsterite interface, catalyzed by the Lewis acid–base behavior of surface O^2−^ and Mg^2+^ sites.^19^ Similarly, calculations have shown that the spectroscopic features of the molecular species upon interaction with forsterite are heavily influenced by its complex surface morphology.^20^ Thus, modeling atomistically a sufficiently large pool of adsorption structures in different coordination environments on the silicate surfaces is key to obtain an accurate representation of the potential vibrational modes in the laboratory and, ultimately, to unravel the true nature of the interaction between molecules and these mineral surfaces in the interstellar and circumstellar environments.

Among the most spectroscopically relevant astrochemical molecules, CO_2_ stands out due to its observed abundance in interstellar ices, ranging from 10 to 23% relative to H_2_O in dense molecular clouds and forming segregated CO_2_-rich ice phases upon thermal processing of the grain.^21^ Given the affinity of CO_2_ towards forsterite surfaces,^22^ the adsorption of CO_2_ and incorporation into the mineral parent body of interstellar dust particles as carbonates has been postulated to account for the anomalous oxygen depletion in the denser ISM.^23,24^ Furthermore, the interaction of CO_2_ and silicate-bearing meteoritic and volcanic particles has shown to be essential for the formation of key precursors in the abiotic synthesis of relevant biomolecules on early-Earth environments.^25^

In a similar context, SO_2_ is a key sulfur-bearing molecule in the ISM. Jointly with OCS, they are both major carriers of gaseous sulfur and are the only sulfurated molecules detected in interstellar ices to date.^26,27^ SO_2_ adsorption and subsequent incorporation into silicate samples is of most interest given the long-standing problem of sulfur depletion in the gas phase.^12,28^ Previous analyses on the adsorption of SO_2_ on MgO surfaces have shown an efficient activation of SO_2_ onto terminal lattice O^2−^ sites,^29,30^ hinting at the potential role of acid and basic sites of forsterite in similar mechanisms.

**Fig. 1:**
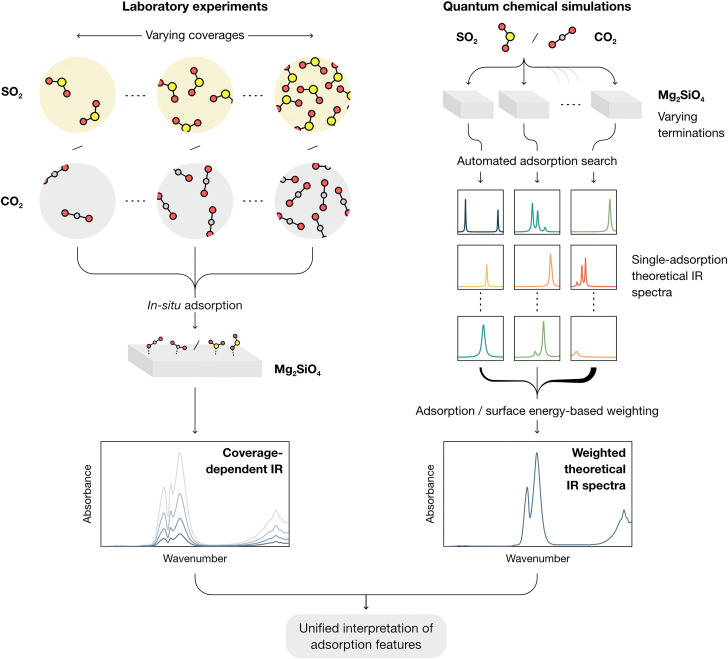
Scheme of the combined framework presented in this work, by which the adsorption features of the studied molecules, SO_2_ and CO_2_, on forsterite, are studied by: *in situ* adsorption IR spectra at different surface coverages both on amorphous and crystalline forsterite; and an automated computational approach that models all potential adsorptions on the theoretical terminations of crystalline forsterite, and aggregates the individual spectra of adsorption configurations weighed on an adsorption energy-based Boltzmann distribution and a surface energy factor. Ultimately, this workflow provides a complete understanding of the adsorption features on the studied interface.

In this work, a combined framework for a comprehensive study of the interactions of CO_2_ and SO_2_ with forsterite surfaces (here used as a test case for cosmic silicates) is showcased, as schematized in Fig. 1. From the experimental side, molecular adsorption is studied by *in situ* IR spectroscopy at progressively declining coverages both on amorphous (AMS) and crystalline (CMS) forsterite at 100 K, akin to the conditions found in collapsing dense molecular clouds and cometary grains. From the modelling side, an automated computational approach is followed to obtain all potential adsorption structures of both molecules onto all theorized forsterite terminations, aimed at representing the local chemical environments found both in crystalline and amorphous surfaces. This approach simplifies the treatment of the amorphous forsterite, whose atomistic structure is unknown and would require modelling large and amorphized unit cell as recently studied by some of us.^31^ A termination-factored adsorption energy-based Boltzmann distribution is then utilized to obtain total weighed spectra of the interaction of SO_2_ and CO_2_ and forsterite (ensuring that the final theoretical IR spectra reflect the complexity of dust grain interfaces under astrophysical conditions), which are compared with the experimental measurements.

## Methods

### Experimental details

#### Materials

Amorphous magnesium silicate (Mg_2_SiO_4_, AMS) was prepared by Yamamoto *et al.* through the evaporation of Mg(OH)_2_ and SiO_2_ precursors in a high temperature plasma (∼10 000 K) and following quenching.^32^ Its specific surface area was 37 m^2^ g^−1^.

Crystalline magnesium silicate (Mg_2_SiO_4_, CMS) was prepared from AMS by heating it at 1073 K for 24 h in air. Its specific surface area was 26 m^2^ g^−1^.

#### IR spectroscopy

The samples were pressed in the form of self-supporting pellets, suitable for transmission IR measurements, and placed in a quartz low-temperature infrared cell equipped with KBr windows, directly connected to a conventional vacuum line, which allows performing all thermal treatments and adsorption–desorption experiments *in situ*.^33^ Prior to the measurements, the pellets were activated at 400 °C for 1 hour under vacuum for cleaning the materials surfaces from undesired adsorbed molecules, mainly atmospheric H_2_O molecules. The CO_2_ and SO_2_ adsorption were monitored using a Bruker Equinox 55 spectrometer equipped with a DTGS detector at 4 cm^−1^ resolution and averaging 128 scans to obtain a good signal/noise ratio. The activated samples were cooled down for 10 minutes by the admission of liquid-N_2_ into the IR cell reservoir reaching a *T* ∼ 100 K. Subsequently, 5 mbar of CO_2_ or SO_2_ were admitted inside the IR cell, then the temperature was freely increased by the natural liquid-N_2_ evaporation until room temperature producing a progressive desorption of the selected molecules from the materials surfaces.

The spectrum of the sample before the adsorbate admission was subtracted to the whole batch of subsequent spectra. The spectra were also normalized by the optical density (weight of the of pellet in mg/area of the pellet in cm^2^) to normalize for the differences in the thickness of the pellets.

### Computational details

#### Generation and optimization of Mg_2_SiO_4_ slabs

Periodic density functional theory (DFT) calculations were performed using the Vienna Ab Initio Simulation Package (VASP) code, version 6.3.2.^34^ The bulk structure of orthorhombic forsterite (Mg_2_SiO_4_), belonging to the Pbnm space group, was obtained through the Materials Project^35^ (id mp-2895), and was optimized using the Perdew–Burke–Ernzerhof (PBE) functional,^36^ with a plane wave energy cut-off of 500 eV and a force-based convergence criterion of 0.015 eV Å^−1^. Equilibrium lattice constants were obtained by extracting the minima of the Birch–Murnaghan equation of state of the energy of the unit cell against its volume, which was adjusted by a factor ranging from 0.98 to 1.02, incrementing in steps of 0.01. This yielded a cell with *a*, *b* and *c* lattice constants of 4.779 Å, 6.017 Å and 10.271 Å, respectively. During lattice optimization, sampling of the first Brillouin zone was performed using *Γ*-centered *k*-point grids of 2 × 2 × 1, 4 × 4 × 2, 8 × 6 × 4 and 12 × 10 × 6. The 4 × 4 × 2 *k*-point grid was selected following an energy convergence criterion of 1 meV atom^−1^, corresponding to a mean *k*-point density of 21.24 *k*-points/Å^−1^ along all axes. This setting was used in all subsequent DFT calculations, unless stated otherwise.

Surface models of forsterite were built using PolyCleaver,^37^ an in-house algorithm that generates charge-neutral and non-polar slabs from crystalline bulks of ionic compounds with polyatomic anions. Hence, our base systems were modelled as slabs with thicknesses of *ca.* 15 Å (value above which surface energy became invariable) and containing the most energetically favorable Miller indices predicted for forsterite,^17,37,38^*i.e.*, (001), (010), (021), (101), (110), (111) and (120) (note that indices in the frequently used Pbnm space group can be converted to the standard *Pnma* space group by exchanging the last two indices). A vacuum of at least 15 Å was included in the *c* axis to avoid spurious interactions within the direction perpendicular to the plane. Atomic positions were optimized by considering a free-standing slab model, where all atoms were allowed to relax while keeping the unit cell parameters fixed at the bulk values. Surface energies, as well as the fraction of each termination in the equilibrium shape of forsterite using a Wulff construction method, were taken from previous work;^37^ these values are reported in Table S1 (ESI†).

#### Active surface site identification and molecular adsorption sampling

Surface sites on the modelled slabs of forsterite were identified through a Delaunay triangulation of surface atomic centers, as described elsewhere.^39^ In this framework, points at the vertices, edge midpoints and centers of the generated triangles were deemed to represent surface atop, bridge and fcc/hcp sites akin to those found on conventional metallic surfaces. In contrast with literature, where determination of surface atoms is performed *via* height-based criteria (*e.g.*, all atoms below 0.5 Å from the highest atom are deemed as surface species), the roughness of the studied surfaces (especially those of high Miller indexes) yields surface atoms with very diverse *z* coordinates, and thus requires a different approach. Hence, identification of those atoms which belong to the surface is done through a systematic procedure: a given atom will be classified as superficial if it is above all atoms around it, *i.e.*, if the coordinates of its atomic center are not eclipsed in the positive *c* direction by any circle defined by all other atomic center coordinates plus the ionic radii of the atoms they define. A *K*-dimensional tree was constructed from the coordinates of all calculated potential surface adsorption sites and was used iteratively to calculate all pairs of points within 0.7 Å along the *ab* plane and replace them by their centroid, until all points were separated by a distance above the threshold value. This was to both reduce dimensionality of the adsorption space and to avoid any group of closely neighboring points describing the same potential adsorption site.

The adsorption of CO_2_ and SO_2_ was investigated on the unit cell of the modelled slabs of forsterite on supercells with *p*(1 × 2) or *p*(2 × 1) multiplicity to ensure all slabs were close in size for consistency and avoid spurious interactions of the adsorbates with neighboring images. Then, molecule–surface interaction was assessed using a procedural approach: first, for each predicted adsorption site point, the molecule was positioned on the *xy* coordinates of the point and 3 Å away from the surface along the *z* coordinate. Then, the molecule was rotated and approached to the surface through a given anchor point until contact (defined as overlapping of the covalent radii of the atomic species of the molecule and of the surface, at which point was then separated by 0.5 Å to remove any steric hindrance and allow any diffusion to a neighboring stable position). This was carried out for one anchor point per molecule (O in both CO_2_ and SO_2_). The initial guess geometry of the approached molecule on the slab was then optimized using the semiempirical GFN1-xTB tight binding method implemented for periodic systems in the tblite package,^40^ keeping the atoms of the slab fixed to limit the drawbacks of the GFN1-xTB method with highly ionic surfaces. The Broyden–Fletcher–Goldfarb–Shanno (BFGS) algorithm implemented in the atomic simulation environment package (ASE)^41^ was used for structural optimization with a convergence criterion of 0.001 eV Å^−1^. This method allows us to rapidly screen how the molecules interact with the surface at each adsorption site and potentially migrate to a neighboring site, yielding geometries akin to those obtainable using DFT. Nonetheless, an initial assessment of the method revealed that GFN1-xTB often encountered structural global minima corresponding to bidentately chemisorbed molecules which are then found to be higher in energy than their respective monodentate counterparts using DFT. To ensure that our initial screening did not fall in spurious energy pitfalls induced by this method, an additional per-atom constraint was introduced where an external Hookean-type repulsive force of 15 eV Å^−2^ was applied between surface and adsorbate atoms within a distance cut-off of 2.4 Å. To account for structures converging to the same highly stable adsorption site, final geometries were then passed through a filter to remove similar structures within a root-mean-square deviation (RMSD) threshold of 2 Å. After careful inspection of the final optimized structures, this automated pipeline allows us to obtain geometrically accurate initial candidates with minimal user intervention, upon which high-precision optimizations at the DFT level of theory will be subsequently applied, as detailed below.

Finally, the positions of all atoms in the filtered geometries were optimized at the PBE level using the residual minimization method with direct inversion in the iterative subspace (RMM-DIIS) algorithm^42^ to ensure geometrical accuracy and replicability. Long-range interactions were considered by using Grimme's D2 dispersion model^43^ for all atoms, including the modified parameters for Mg^2+^ outlined in the revised D*(*N*) model to take into account its highly ionic character.^44^ At variance with surface relaxation, optimizations of the molecule adsorptions were performed at Γ-point only; this method yielded sufficiently accurate geometries while limiting the computational cost, given the large amount of molecular adsorptions modelled. To prevent spurious molecular dissociation of some SO_2_ molecules observed in preliminary DFT calculations (where the GFN1-xTB method yielded adsorbates too close to the surface), the *z* distance of the adsorbate atoms in the filtered geometries that showed that behavior with respect to the surface was increased by 1.5 to 2.5 Å prior to relaxation with DFT. In these latter cases, a blocked-Davidson iteration scheme was used for optimization.

Adsorption energies (Δ*E*_B_) of the optimized molecule–surface systems were calculated as:Δ*E*_B_ = *E*_*i*_ − *E*^*m*^_slab_ − *E*_mol_where *E*_*i*_ is the total energy of the molecule–surface adsorption complex *i*, *E*^*m*^_slab_ is total energy of the isolated slab, and *E*_mol_ is the total energy of the isolated molecule in the gas phase. With the above definition, negative Δ*E*_B_ values result in favorable adsorption. 3D representations of representative adsorbates were rendered with the OVITO software.^45^

#### Simulation of multiconfigurational infrared spectra

To model the infrared (IR) spectra of the molecules adsorbed on the forsterite surfaces, *Γ*-point vibrational frequencies and Born effective charges were calculated using density functional perturbation theory (DFPT), in a routine implemented in the VASP code. It is important to note that while the Hessian matrix is computed using a molecular fragment, the associated energy variations and gradients due to nuclear displacements account for the entire electronic response of the system. This ensures that all electronic charge redistributions are included in the fragment calculation. Mechanical coupling with the rest of the structure, into which the fragment is embedded, is indeed neglected, but the computed vibrational frequencies and IR intensities are minimally affected, since the interaction of the ad-atoms with the phonon modes of the mineral surface are very weak. Vibrational intensities were calculated by the approach described by Gianozzi and Baroni,^46^ as implemented in the VASP-infrared-intensities script.^47^ To account for any systematic errors in the adopted functional and anharmonicity, adsorbate vibrational harmonic frequencies involving the adsorption of CO_2_ were scaled by a factor of 0.997, corresponding to the difference of the largest IR-active stretching vibrational mode of ^12^C^16^O_2_ calculated with PBE (2356 cm^−1^) with respect to tabulated data^48^ (2349 cm^−1^); similarly, frequencies of the adsorbed SO_2_ molecule were scaled by a factor of 1.067, corresponding to the difference between the largest calculated ^32^S^16^O_2_ molecule stretching mode (1277 cm^−1^) compared to tabulated data (1362 cm^−1^). The theoretical infrared spectra for CO_2_ and SO_2_ are obtained by summing Lorentzian-broadened vibrational peaks at full width at half maximum of each individual adsorption. The absolute intensity of each peak is weighted by a Boltzmann factor, accounting for the relative stability of each adsorption with respect to the least energetic configuration on a given termination, plus a surface factor, representing the fractional proportion of each surface termination in the equilibrium shape obtained by the Wulff construction method. The final IR spectrum, *S*, is given by:
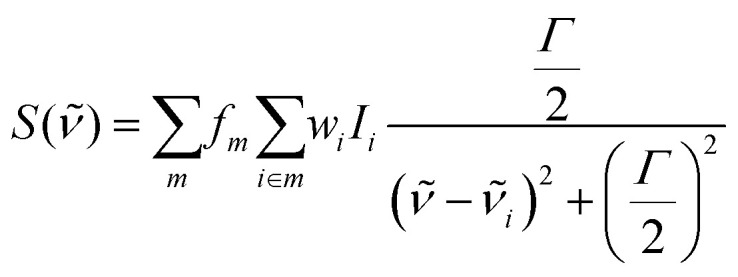
where *f*_*m*_ is the surface factor for the surface with Miller index *m*, *I*_*i*_ and *ν̃*_*i*_ are the unweighted intensity and the wavenumber (in cm^−1^) of the theoretical IR peak of adsorption mode *i*, respectively, *Γ* is the Lorentzian broadening parameter (which was conservatively set to 15 cm^−1^), and *w*_*i*_ is the weighted Boltzmann factor, defined as:
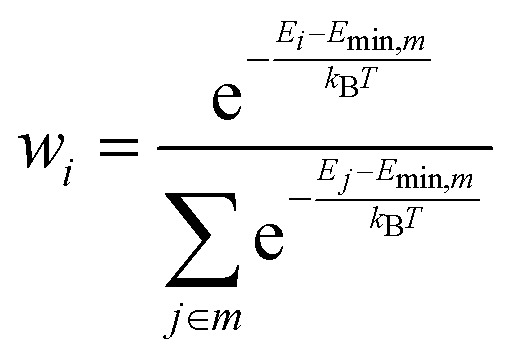
where *E*_min,*m*_ is the energy of the most stable adsorption on each surface termination with Miller index *m* and *k*_B_ is the Boltzmann constant. The temperature *T* is set to the experimental value of 100 K. Final spectra are normalized.

## Results and discussion

### IR spectroscopy

**Fig. 2:**
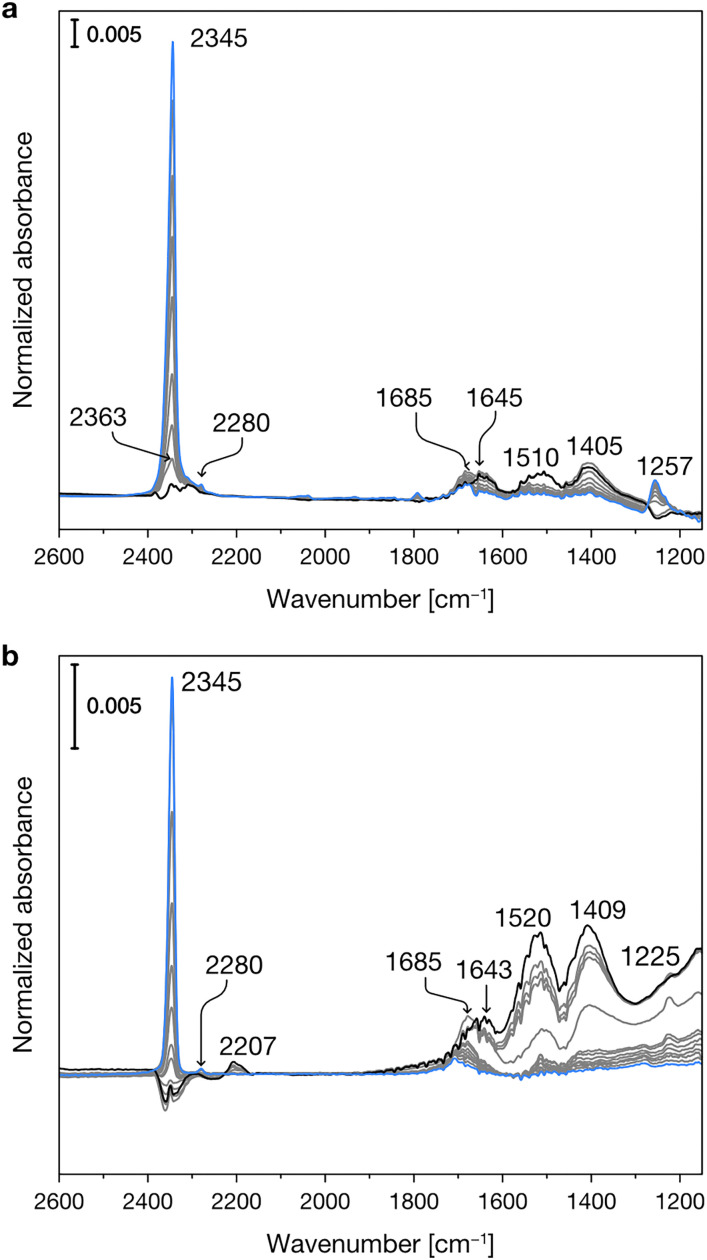
Experimental IR spectra of CO_2_ adsorbed at progressively increasing temperatures (from blue to black spectra) on crystalline (a) and amorphous (b) Mg-silicates previously outgassed at 673 K.

Fig. 2 reports the experimental IR spectra of CO_2_ adsorption on the dehydrated Mg-silicates. In both samples, in the initial stages of the adsorption at low temperature (blue spectra), the most intense peak is centered at 2345 cm^−1^ and can be assigned to weakly adsorbed CO_2_.^49,50^ During the temperature increase (see experimental section), we observe the progressive desorption of the physisorbed CO_2_ and the parallel growth of new bands in the 1700–1200 cm^−1^ spectral region, which are due to the formation of surface carbonates.^49,50^

**Fig. 3:**
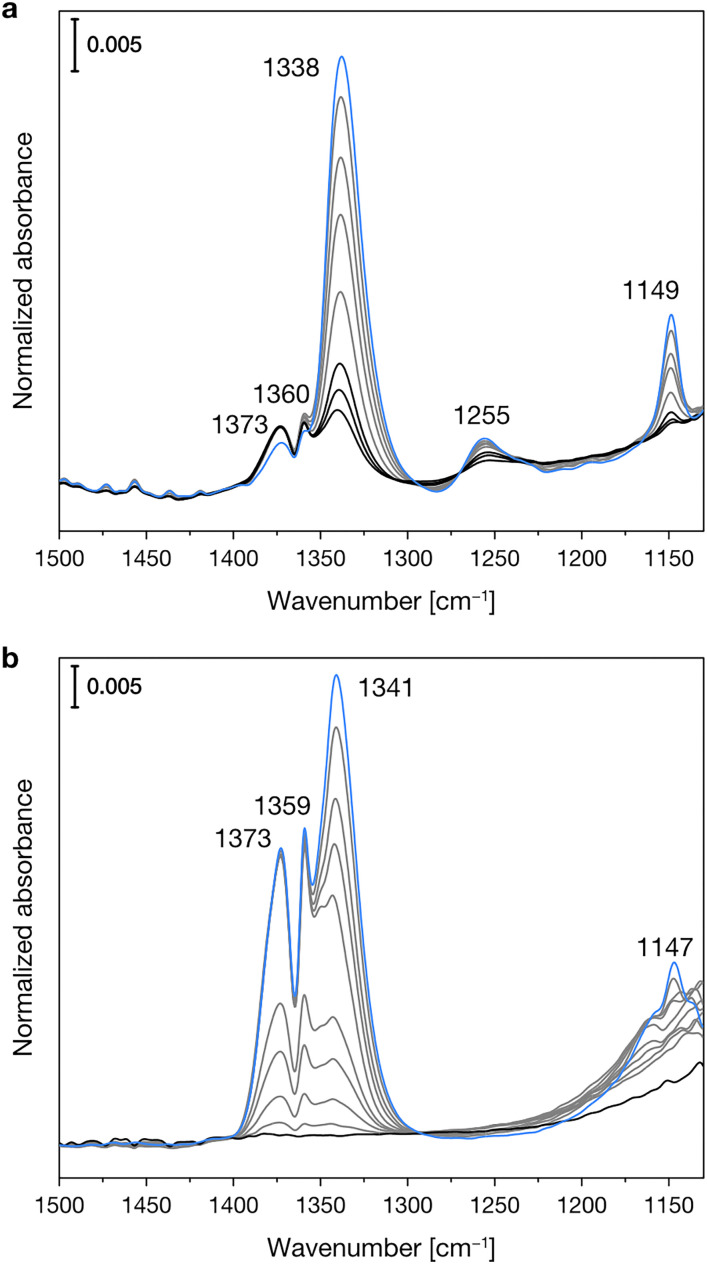
Experimental IR spectra of SO_2_ adsorbed at progressively increasing temperatures (from blue to black spectra) on crystalline (a) and amorphous (b) Mg-silicates previously outgassed at 673 K.

Fig. 3 shows the experimental IR spectra of SO_2_ adsorption on the dehydrated Mg-silicates. For both amorphous and crystalline materials, the main band is centered at *ca.* 1340 cm^−1^ and can be ascribed to the asymmetric stretching mode of adsorbed SO_2_. The slightly higher wavenumber observed for the amorphous sample (1341 *vs.* 1338 cm^−1^) can be related to its higher surface basicity.^51^ Around 1150 cm^−1^, we can note the presence of the corresponding symmetric stretching mode. Finally, we can observe signals between 1375 and 1360 cm^−1^, which have been attributed to the coexistence of SO_2_ in both the liquid-like and gas phases.^52^ The different intensities of these signals in the crystalline and amorphous Mg-silicates are likely due to their differing porosity.

### Computational results

**Fig. 4:**
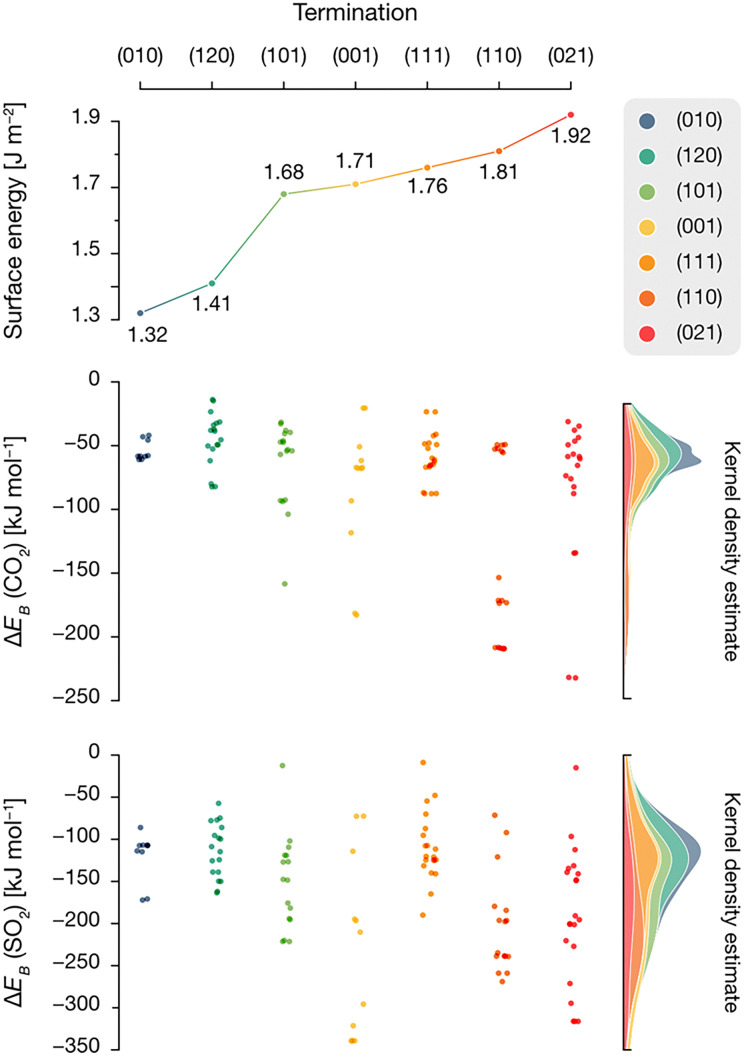
Surface energy of the calculated baseline terminations (top panel), along with adsorption energy distribution of CO_2_ and SO_2_ on each surface. Each point denotes a unique adsorption on each termination, as indicated by their color code. A kernel density estimate distribution is included denoting the range of energies where adsorptions are scattered on each surface.

**Fig. 5:**
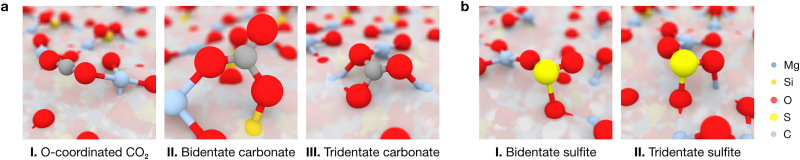
Atomic models of representative adsorption modes of (a) CO_2_ and (b) SO_2_ on the forsterite surfaces. Panel (a) depicts the most stable adsorptions on the (120), (101) and (021) surfaces, corresponding to monodentate CO_2_ interacting with a Mg^2+^ site through a terminal O atom, or forming bidentate and tridentate surface carbonates, respectively. Panel (b) depicts the most and eight-most stable SO_2_ adsorptions on the (120) surface, yielding to the formation of bidentate or tridentate surface sulfites. Sub-surface atoms have been whitened out for clarity.

To obtain a complete picture of the binding modes of CO_2_ and SO_2_ on the forsterite terminations, we assessed the interactions of both molecules with the seven characteristic forsterite terminations present in the crystalline shape of the crystal at the PBE-D*(*N*) level (see Methods section). Fig. 4 shows all studied adsorption configurations based on their computed adsorption energy, arranged from left to right by increasing surface energy of their corresponding forsterite termination; the position of the corresponding optimized adsorbates on the surfaces is displayed in Fig. S1 (ESI†). Generally, this analysis revealed that the surface energy of the bare surface is inversely correlated with their binding potential: more stable terminations—those with lower surface energies, such as the (010) or the (120) surfaces—have a lower adsorption energy towards both CO_2_ (middle panel) and SO_2_ (lower panel) than high surface energy terminations such as (110) or (021), whose adsorbates possess more favorable adsorption energy values. This is, in part, due to the nature of both CO_2_ and SO_2_, and the surface atoms of every surface; CO_2_ has been shown to spontaneously activate on the surface basic O^2−^ sites of forsterite,^22^ effectively forming carbonate units with surface O ions. In our analysis, this predominantly occurs on higher surface energy terminations, accounting for the low adsorption energy structures presented in the middle panel of Fig. 4. This results in two main energy differentiated regimes for CO_2_ adsorption: it either adsorbs through a terminal O atom on acidic Mg^2+^ sites with adsorption energies of up to −100 kcal mol^−1^, or it activates on the surface upon interaction of its C and O atoms with an exposed O^2−^ ion and either one or two Mg^2+^ sites (depending on the steric availability), forming bidentate or tridentate bound carbonates with adsorption energies of −100 to −200 kcal mol^−1^ (Fig. 5a). The former configuration conforms the largest population of adsorbates on all surfaces, as shown in the kernel density distribution (see Fig. 4).

In contrast to the non-polar CO_2_, the polar nature of SO_2_ as a bent geometry facilitates its interaction with surface O^2−^ sites which, sterically, are unavailable for CO_2_ activation. Hence, SO_2_ adsorbates on the studied forsterite terminations display strikingly more favorable adsorption energies, up to −340 kcal mol^−1^; more importantly, we see that the energetic distribution of the adsorbates is spread out across all adsorption energy values, yielding a broader kernel density distribution. This is partly due to SO_2_ interacting similarly in all simulations, *i.e.*, S interacts with a surface O^2−^ site and one or both of the O atoms of SO_2_ bind to surface Mg^2+^ sites, forming bidentate or tridentate bound sulfites, analogous to its behavior on stepped MgO surfaces^29^ (Fig. 5b).

**Fig. 6:**
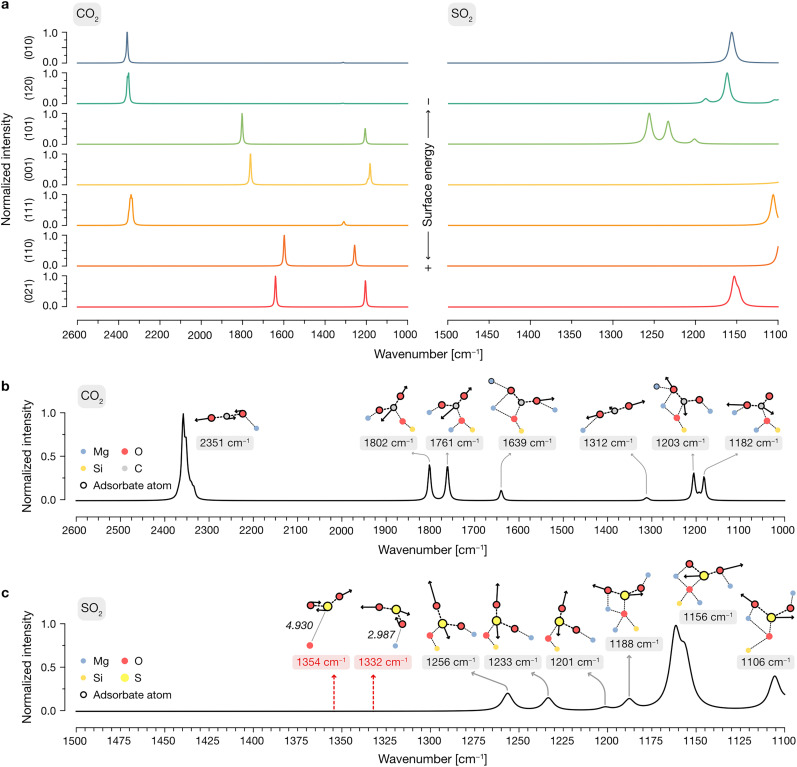
(a) IR spectra of the adsorbed CO_2_ and SO_2_ molecules on the modelled terminations of forsterite. Spectra are arranged from top to bottom with respect to the surface energy of their respective surface termination. All spectra contain the peaks arising from a Boltzmann distribution of the adsorptions on the surface, where the reference value is selected as the most stable adsorption, as described in the Methods section. (b) Total IR spectrum of CO_2_ considering all adsorption sites, adsorption configurations and surface terminations (see Methods). Representations of the atomic displacements within the vibrational modes corresponding to the theoretical peaks in the IR spectrum are included as insets. In these, atomic displacements within the vibrational mode are represented as black arrows with scaled magnitudes, and atoms belonging to the adsorbate are outlined. Atomic bonds are depicted as dotted lines. (c) Total IR spectrum of SO_2_, in a similar fashion to (b). Schematic representations for calculated vibrational modes corresponding to non-adsorbed and physisorbed SO_2_ on the forsterite are included and labelled with a reddish background; these features are present in the high-coverage experimental regime but contain a negligible weight in the Boltzmann energy distribution in the calculations, and thus display no associated peaks in the global theoretical IR spectrum. Relevant distances between the SO_2_ molecule and the surface, in Å, are depicted in italics.

To elucidate the vibrational modes governing the spectroscopic features observed experimentally, we then generated per-surface IR spectra including all adsorptions of SO_2_ and CO_2_, as displayed in Fig. 6a. In these spectra, a weight was applied to the calculated IR intensities of each configuration following a Boltzmann distribution based on their relative adsorption energies, as detailed in the Methods section. This procedure is aimed at representing a low-coverage situation in which the contribution of each adsorbate structure to the simulated total IR spectra is determined by the probability of an adsorbate to bind to that respective site in each orientation.

In the case of CO_2_, this approach yields very different features depending on the surface: given that the low surface energy terminations cannot activate CO_2_ due to their peculiar exposed O^2−^/Mg^2+^ site distribution (see above), the predominant features present in the adsorbate spectra of the (010), (120) and (111) are those of the gas-phase CO_2_, arising from physisorbed molecules on the surface with relatively low favorable adsorption energies. In contrast, given the ability of the (101), (001), (110) and (021) surfaces to activate CO_2_ with lower adsorption energies than its physisorbed counterpart, any contributions of the latter to the global IR are wiped away by the Boltzmann factor by those of newly conformed carbonate structures on the surface.

After determining the predominant IR features on each forsterite termination, we then proceeded to build a global spectrum where the contribution of each surface to the crystalline equilibrium shape (Table S1, ESI†) is computed. These global spectra are depicted in Fig. 6b and c for CO_2_ and SO_2_, respectively, displaying representative vibrational modes contributing to each theoretical peak.

Our simulations show that the IR features arising from low surface energy terminations such as (010) and (120) dominate the spectrum: we predict the characteristic peak corresponding to the asymmetric stretching of CO_2_ to appear prominently at 2351 cm^−1^, indicative of weakly physisorbed CO_2_. The slight redshift of this mode relative to gas-phase CO_2_ (2349 cm^−1^) is due to the interaction of a terminal O with a surface Mg^2+^ site; this interaction breaks the symmetry of the adsorbed molecule, rendering the normally IR-inactive symmetric stretching mode weakly activated at a frequency of 1312 cm^−1^. We assign all other calculated features to vibrational modes associated with the formation of surface-bound carbonates, with frequencies of 1802, 1761, 1639, 1203 and 1182 cm^−1^. In these configurations, CO_2_ binds either in a bidentate fashion, with its C and one O atom interacting with surface O^2−^ and Mg^2+^ sites, respectively, or in a tridentate mode, where the second terminal oxygen of CO_2_ also coordinates to a nearby Mg^2+^ site.

For SO_2_, our simulations do not predict the existence of gas-phase features in the most stable adsorption configurations on each termination, as any peaks arising from weakly physisorbed molecules have a negligible impact in the computed Boltzmann distribution due to their high adsorption energies compared to those coming from the chemisorbed SO_2_. Upon building the global IR spectrum, our calculations predict the modes of these weakly adsorbed molecules to be redshifted relative to gas-phase SO_2_ due to their interaction with the surfaces, as exemplified in the representative red-labeled modes in Fig. 6c. Thus, all modes present in the global IR spectrum are assigned to chemisorbed SO_2_ which, akin to CO_2_, binds to surface O^2−^ and Mg^2+^ sites in a bidentate fashion through its S and a terminal O atom, respectively, or in a tridentate fashion where the remaining O binds to a neighboring Mg^2+^ site. The resulting features in the IR spectrum span a range of frequencies, with increasing coordination strength leading to more pronounced redshifts: while bidentate bound molecules yield peaks at 1256, 1233 and 1201 cm^−1^, the strongest redshifts are associated with tridentate binding, producing peaks at 1188, 1156, and 1106 cm^−1^. Notably, the peak at 1156 cm^−1^, corresponding to the “symmetric” stretching of the tridentate SO_2_, dominates the spectrum due to its association with the most thermodynamically favored adsorption configurations on the low surface energy (010) and (120) terminations.

### Integrating experiments and theory

A comparison between experimental results and the simulated global IR spectrum for CO_2_ and SO_2_ reveals good agreement in both the position and evolution of key spectral features (as shown in Fig. S2 (ESI†) for the case of CMS as an illustrative case). In the case of CO_2_, low temperature experimental IR spectra are dominated by a peak at 2345 cm^−1^, progressively diminishing with increasing temperature and giving rise to new absorptions between 1700 and 1200 cm^−1^, consistent with a predominance of carbonates at low coverages. In the experimental spectra, the carbonate signals become more evident at higher temperatures because their formation requires overcoming a relatively high activation barrier to bend the CO_2_ molecule. This aspect is not considered in the theoretical part, in which the adsorbed species are already prepared to be ready for carbonate formation. These trends are present in the theoretical spectrum: while the contributions from low-energy terminations—particularly (010) and (120)—account for the physisorbed CO_2_ signal, higher surface energy terminations contain peaks ranging from approximately 1800 to 1200 cm^−1^. While our calculations do not account for the formation of additional carbonate layers at higher CO_2_ coverages, nor for the interaction of CO_2_ with traces of co-adsorbed water—which may contribute to the observed discrepancies in carbonate peak positions—the theoretical results suggest that as the most favorable adsorption sites become occupied, configurations with higher adsorption energies begin to dominate. This leads to an increased contribution from carbonate-like modes, which progressively overshadow the spectral features associated with physisorbed CO_2_ in the high temperature regimes.

In the case of SO_2_, our simulations accurately predict the presence of chemisorbed structures, characterized by the peak located around 1150 cm^−1^ both in theory and experiments. Our calculations predict this peak to be prominent under both high and low coverage conditions: at high coverages (low temperature), we expect it to arise from the symmetric stretching mode of weakly physisorbed SO_2_ and from the modes of surface-bound species, whereas at low coverages (high temperature), it originates exclusively from chemisorbed SO_2_ molecules predicted by the simulations. Additionally, both the experimental broad bands and the peak observed at 1255 cm^−1^ in the CMS samples are consistent with the simulated results, which suggest a diverse landscape of interaction modes that drastically affect the spectroscopic features. Illustrative representations of the atomic configurations conforming the most stable adsorptions of CO_2_ and SO_2_ on each surface termination are shown in Fig. S3–S16 (ESI†).

## Conclusions

In this work, we have combined *in situ* IR spectroscopy and an automated computational framework to systematically investigate the adsorption behavior of CO_2_ and SO_2_ on amorphous and crystalline forsterite surfaces under astrophysical relevant conditions. Our results reveal that both molecules exhibit distinct spectroscopic signatures depending on their interaction strength with the surface, the surface termination, and the coverage regime.

By explicitly accounting for the relative stability of adsorption modes *via* Boltzmann statistics and the morphological distribution of surface terminations in the equilibrium shape of the crystal, our theoretical approach achieves good agreement with experimental IR spectra. This methodology not only enables the assignment of vibrational features to specific surface configurations, but also provides a framework to study the spectroscopic behavior of other interstellar molecules on mineral surfaces of cosmic relevance.

## Conflicts of interest

There are no conflicts to declare.

## Supplementary Material

CP-027-D5CP01699A-s001

## Data Availability

The data supporting this article, including supporting figures and tables, have been included as part of the ESI.† All computational data presented herein, including the Jupyter notebooks for the generation of weighed theoretical IR spectra and scripts for the automatic optimization of adsorbates on the silicate surfaces, are shared with a GNU General Public License v4.0 license through a Zenodo dataset with the following DOI: https://doi.org/10.5281/zenodo.15341156.

## References

[cit1] Do-Duy T., Wright C. M., Fujiyoshi T., Glasse A., Siebenmorgen R., Smith R., Stecklum B., Sterzik M. (2020). Mon. Not. R. Astron. Soc..

[cit2] Takigawa A., Tachibana S., Nagahara H., Ozawa K., Yokoyama M. (2009). Adv. Pharm. J..

[cit3] De Vries B. L., Min M., Waters L. B. F. M., Blommaert J. A. D. L., Kemper F. (2010). Astron. Astrophys..

[cit4] Draine B. T. , in *Cosmic Dust - Near and Far*, ed. Th. Henning, E. Grün and J. Steinacker, ASP Conference Series, 2009, vol. 414, pp. 453–472

[cit5] Spoon H. W. W., Tielens A. G. G. M., Armus L., Sloan G. C., Sargent B., Cami J., Charmandaris V., Houck J. R., Soifer B. T. (2006). Adv. Pharm. J..

[cit6] Graham A. L., Easton A. J., Hutchison R. (1977). Mineral. Mag..

[cit7] Kołodziej M., Michalska D., Załęski K., Iatsunskyi I., Muszyński A., Coy E. (2025). Sci. Rep..

[cit8] Messenger S., Keller L. P., Stadermann F. J., Walker R. M., Zinner E. (2003). Science.

[cit9] J. Bradley , in Astromineralogy, ed T. Henning, Springer Berlin Heidelberg, Berlin, Heidelberg, 2010, vol. 815, pp. 259–276

[cit10] Nakamura-Messenger K., Keller L. P., Clemett S. J., Messenger S., Ito M. (2011). Meteorit. Planet. Sci..

[cit11] Sierks H., Barbieri C., Lamy P. L., Rodrigo R., Koschny D., Rickman H., Keller H. U., Agarwal J., A’Hearn M. F., Angrilli F., Auger A.-T., Barucci M. A., Bertaux J.-L., Bertini I., Besse S., Bodewits D., Capanna C., Cremonese G., Da Deppo V., Davidsson B., Debei S., De Cecco M., Ferri F., Fornasier S., Fulle M., Gaskell R., Giacomini L., Groussin O., Gutierrez-Marques P., Gutiérrez P. J., Güttler C., Hoekzema N., Hviid S. F., Ip W.-H., Jorda L., Knollenberg J., Kovacs G., Kramm J. R., Kührt E., Küppers M., La Forgia F., Lara L. M., Lazzarin M., Leyrat C., Lopez Moreno J. J., Magrin S., Marchi S., Marzari F., Massironi M., Michalik H., Moissl R., Mottola S., Naletto G., Oklay N., Pajola M., Pertile M., Preusker F., Sabau L., Scholten F., Snodgrass C., Thomas N., Tubiana C., Vincent J.-B., Wenzel K.-P., Zaccariotto M., Pätzold M. (2015). Science.

[cit12] Perrero J., Beitia-Antero L., Fuente A., Ugliengo P., Rimola A. (2023). Mon. Not. R. Astron. Soc..

[cit13] Molpeceres G., Rimola A., Ceccarelli C., Kästner J., Ugliengo P., Maté B. (2019). Mon. Not. R. Astron. Soc..

[cit14] Navarro-Ruiz J., Sodupe M., Ugliengo P., Rimola A. (2014). Phys. Chem. Chem. Phys..

[cit15] Santalucia R., Pazzi M., Bonino F., Signorile M., Scarano D., Ugliengo P., Spoto G., Mino L. (2022). Phys. Chem. Chem. Phys..

[cit16] Vinogradoff V., Leyva V., Mates-Torres E., Pepino R., Danger G., Rimola A., Cazals L., Serra C., Pascal R., Meinert C. (2024). Earth Planet. Sci. Lett..

[cit17] Bancone N., Pantaleone S., Ugliengo P., Rimola A., Corno M. (2023). Phys. Chem. Chem. Phys..

[cit18] Bancone N., Pantaleone S., Ugliengo P., Rimola A., Corno M. (2025). ACS Earth Space Chem..

[cit19] Bancone N., Santalucia R., Pantaleone S., Ugliengo P., Mino L., Rimola A., Corno M. (2024). J. Phys. Chem. C.

[cit20] Zamirri L., Pantaleone S., Ugliengo P. (2019). J. Chem. Phys..

[cit21] Gerakines P. A., Whittet D. C. B., Ehrenfreund P., Boogert A. C. A., Tielens A. G. G. M., Schutte W. A., Chiar J. E., Van Dishoeck E. F., Prusti T., Helmich F. P., De Graauw T. (1999). Adv. Pharm. J..

[cit22] Ermolov Y., Vasilchenko A., Lazorenko G. (2024). Int. J. Mater. Sci..

[cit23] Jones A. P., Ysard N. (2019). Astron. Astrophys..

[cit24] Kemper F., Jäger C., Waters L. B. F. M., Henning Th, Molster F. J., Barlow M. J., Lim T., De Koter A. (2002). Nature.

[cit25] Peters S., Semenov D. A., Hochleitner R., Trapp O. (2023). Sci. Rep..

[cit26] McClure M. K., Rocha W. R. M., Pontoppidan K. M., Crouzet N., Chu L. E. U., Dartois E., Lamberts T., Noble J. A., Pendleton Y. J., Perotti G., Qasim D., Rachid M. G., Smith Z. L., Sun F., Beck T. L., Boogert A. C. A., Brown W. A., Caselli P., Charnley S. B., Cuppen H. M., Dickinson H., Drozdovskaya M. N., Egami E., Erkal J., Fraser H., Garrod R. T., Harsono D., Ioppolo S., Jiménez-Serra I., Jin M., Jørgensen J. K., Kristensen L. E., Lis D. C., McCoustra M. R. S., McGuire B. A., Melnick G. J., Öberg K. I., Palumbo M. E., Shimonishi T., Sturm J. A., van Dishoeck E. F., Linnartz H. (2023). Nat. Astron..

[cit27] Rocha W. R. M., Van Dishoeck E. F., Ressler M. E., Van Gelder M. L., Slavicinska K., Brunken N. G. C., Linnartz H., Ray T. P., Beuther H., Caratti A., Garatti O., Geers V., Kavanagh P. J., Klaassen P. D., Justtanont K., Chen Y., Francis L., Gieser C., Perotti G., Tychoniec Ł., Barsony M., Majumdar L., Le Gouellec V. J. M., Chu L. E. U., Lew B. W. P., Henning Th, Wright G. (2024). Astron. Astrophys..

[cit28] Laas J. C., Caselli P. (2019). Astron. Astrophys..

[cit29] Pacchioni G., Clotet A., Ricart J. M. (1994). Surf. Sci..

[cit30] Rodriguez J. A., Jirsak T., Freitag A., Larese J. Z., Maiti A. (2000). J. Phys. Chem. B.

[cit31] Martínez-González J., Navarro-Ruiz J., Rimola A. (2018). Minerals.

[cit32] Yamamoto D., Tachibana S. (2018). ACS Earth Space Chem..

[cit33] Pavan C., Santalucia R., Escolano-Casado G., Ugliengo P., Mino L., Turci F. (2023). Int. J. Mater. Sci..

[cit34] Kresse G., Furthmüller J. (1996). Phys. Rev. B: Condens. Matter Mater. Phys..

[cit35] Jain A., Ong S. P., Hautier G., Chen W., Richards W. D., Dacek S., Cholia S., Gunter D., Skinner D., Ceder G., Persson K. A. (2013). APL Mater..

[cit36] Perdew J. P., Burke K., Ernzerhof M. (1996). Phys. Rev. Lett..

[cit37] Mates-Torres E., Rimola A. (2024). J. Appl. Crystallogr..

[cit38] Bruno M., Massaro F. R., Prencipe M., Demichelis R., De La Pierre M., Nestola F. (2014). J. Phys. Chem. C.

[cit39] Montoya J. H., Persson K. A. (2017). npj Comput. Mater..

[cit40] Grimme S., Bannwarth C., Shushkov P. (2017). J. Chem. Theory Comput..

[cit41] Hjorth Larsen A., Jørgen Mortensen J., Blomqvist J., Castelli I. E., Christensen R., Dułak M., Friis J., Groves M. N., Hammer B., Hargus C., Hermes E. D., Jennings P. C., Bjerre Jensen P., Kermode J., Kitchin J. R., Leonhard Kolsbjerg E., Kubal J., Kaasbjerg K., Lysgaard S., Bergmann Maronsson J., Maxson T., Olsen T., Pastewka L., Peterson A., Rostgaard C., Schiøtz J., Schütt O., Strange M., Thygesen K. S., Vegge T., Vilhelmsen L., Walter M., Zeng Z., Jacobsen K. W. (2017). J. Phys.: Condens. Matter.

[cit42] Pulay P. (1980). Chem. Phys. Lett..

[cit43] Grimme S. (2006). J. Comput. Chem..

[cit44] Cutini M., Maschio L., Ugliengo P. (2020). J. Chem. Theory Comput..

[cit45] Stukowski A. (2010). Modelling Simul. Mater. Sci. Eng..

[cit46] Baroni S., De Gironcoli S., Dal Corso A., Giannozzi P. (2001). Rev. Mod. Phys..

[cit47] Karhánek D. , Dakarhanek/VASP-infrared-intensities (version v1.0) Zenodo, 2020

[cit48] Shimanouchi T. , Tables of molecular vibrational frequencies, consolidated volume i, National Bureau of Standards, Gaithersburg, MD, 1972

[cit49] Xamena F. X. L. I., Zecchina A. (2002). Phys. Chem. Chem. Phys..

[cit50] Mino L., Cesano F., Scarano D., Spoto G., Martra G. (2019). Res. Chem. Intermed..

[cit51] Waqif M., Saad A. M., Bensitel M., Bachelier J., Saur O., Lavalley J.-C. (1992). Faraday Trans..

[cit52] Nash D. B., Betts B. H. (1995). Icarus.

