# Supplementary material for: Revealing SO2 and CO2 adsorption features on forsterite via IR spectroscopy and automated computational approaches
Source: Phys Chem Chem Phys. 2025 Jun 3;27(24):13124–34. doi: 10.1039/d5cp01699a (PMC12147705; doi:10.1039/d5cp01699a)
Supplement: CP-027-D5CP01699A-s001 [file CP-027-D5CP01699A-s001.pdf]

Supplementary Information (SI) for Physical Chemistry Chemical Physics.
This journal is © the Owner Societies 2025

Electronic Supplementary Information

Revealing SO_2 and CO_2 Adsorption Features on Forsterite via IR Spectroscopy and Automated Computational Approaches

Guillermo Escolano Casado[2†‡], Eric Mates-Torres[1‡], Lorenzo Mino[2], Nadia Balucani[3], Piero Ugliengo[2], Albert Rimola[1*]

[1] Departament de Química, Universitat Autònoma de Barcelona, 08193 Bellaterra, Catalonia, Spain

[2] Dipartimento di Chimica and Nanomaterials for Industry and Sustainability (NIS) Centre, Università degli Studi di Torino, via P. Giuria 7, I-10125, Torino, Italy

[3] Dipartimento di Chimica, Biologia e Biotecnologie, Università degli Studi di Perugia, Via Elce di Sotto 8, I-06123 Perugia, Italy

† Present address: Department of Chemistry and Biochemistry, Mendel University in Brno, Zemedelska 1, CZ-613 00, Brno, Czech Republic

‡ These authors contributed equally to this work

*Correspondence: eric.mates@uab.cat, lorenzo.mino@unito.it, albert.rimola@uab.cat

Table S1. Miller indices in the Pbnm and standard Pnma notations of the forsterite space group, along with the surface energies at the PBE-D3 level and the fraction that each termination occupies on the crystal equilibrium shape of forsterite obtained with the Wulff construction method.

Miller index (Pbnm)	Miller index (Pnma)	Surface energy (J m^{-2})	Equilibrium shape fraction
(010)	(001)	1.32	**0.198**
(120)	(102)	1.41	**0.331**
(101)	(110)	1.68	**0.153**
(001)	(010)	1.71	**0.155**
(111)	(111)	1.76	**0.115**
(110)	(101)	1.81	**0.000**
(021)	(012)	1.92	**0.047**

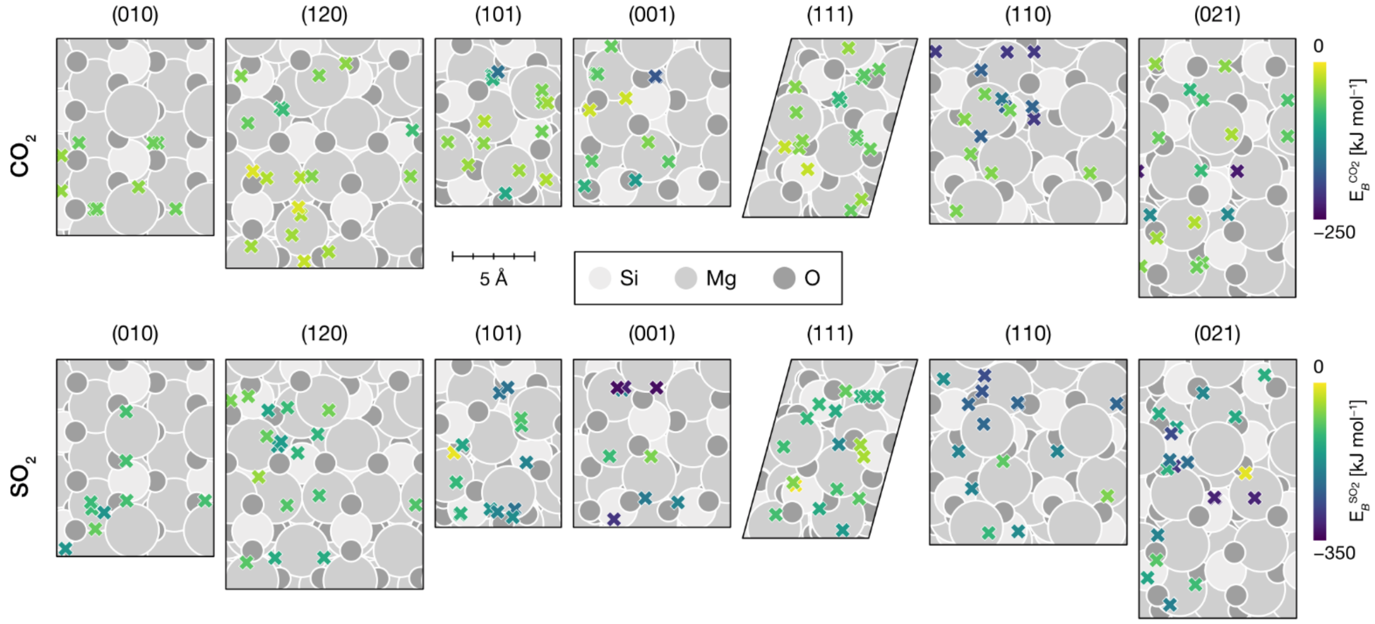

Figure S1. Top view of the surface unit cells depicting the molecular adsorption sites for CO_2 and SO_2. Adsorption sites are denoted as *x* symbols on the surface and correspond to the XY coordinates of the center of mass of the adsorbate in its final, optimal position at the DFT level. Atoms belonging to the surface have been colored in grayscale for readability.

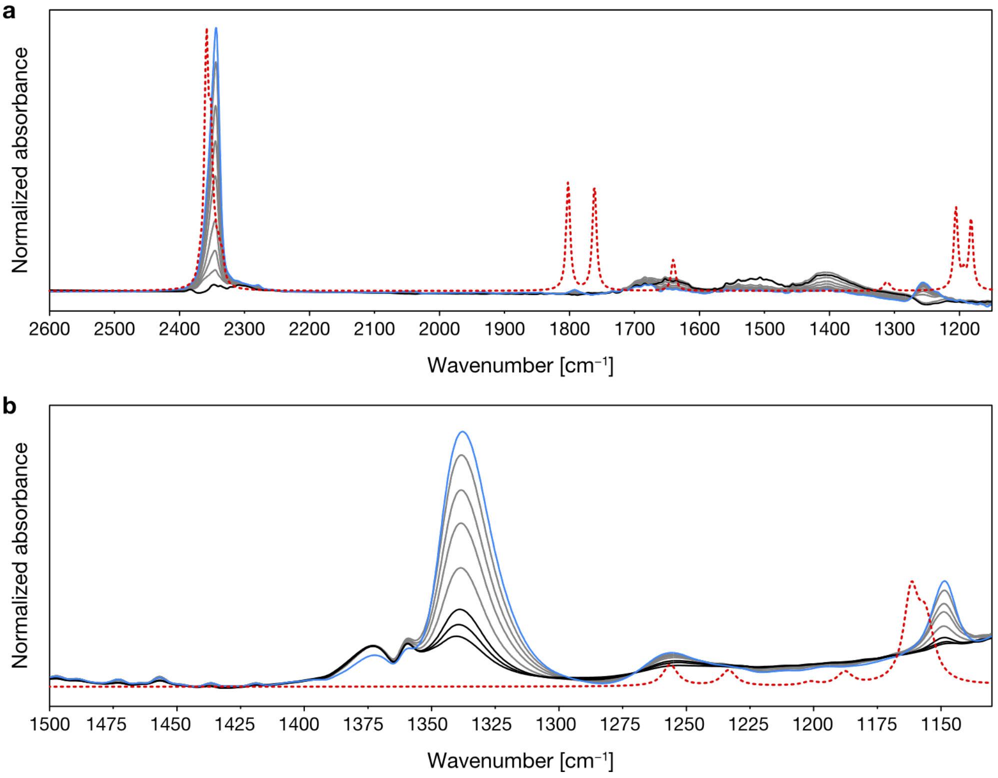

Figure S2. Experimental IR spectra of a) CO_2 and b) SO_2 on crystalline Mg-silicate at progressively increasing temperatures (from blue to black lines); the Boltzmann and Wulff-weighed theoretical total IR spectra are added as dotted red lines for direct comparison.

(010)

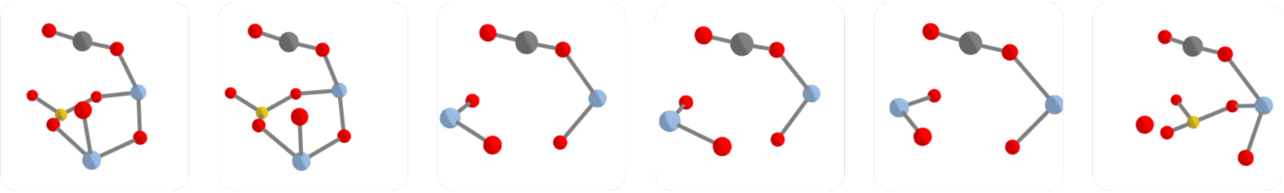

-60.1		-59.98		-57.64		-57.47		-57.44		-57.01	
2358	1.000	2359	1.000	2371	1.000	2369	1.000	2366	1.000	2359	1.000
1312	0.024	1313	0.023	1324	0.011	1323	0.008	1322	0.007	1312	0.020
606	0.087	606	0.086	606	0.081	599	0.096	599	0.094	606	0.092
579	0.102	580	0.100	586	0.054	577	0.066	578	0.065	591	0.082
195	0.004	195	0.004	151	0.005	149	0.004	149	0.004	193	0.004
113	0.001	119	0.001	107	0.001	129	0.001	130	0.001	104	0.001
96	0.000	97	0.000	92	0.000	106	0.000	115	0.000	93	0.000
60	0.001	65	0.003	49	0.000	76	0.000	75	0.001	58	0.003
0	0.003	28	0.002	38	0.001	46	0.002	49	0.002	20	0.002

Figure S3. Atomic representations of the 6 $\mathbf{CO_2}$ adsorption configurations displaying the lowest adsorption energies on the **(010)** surface of Mg_2SiO_4 in our calculations. Only atoms within a radius of 4 Å from the C center are displayed. Adsorption energies are denoted in purple, in kJ mol^{-1}. The arising spectroscopic features from these adsorptions are displayed under the adsorption energy, where the first column is the wavenumber in cm^{-1}, and the second column is the normalized intensity. Blue, dark yellow, red and gray balls represent Mg, Si, O and C atoms, respectively.

(120)

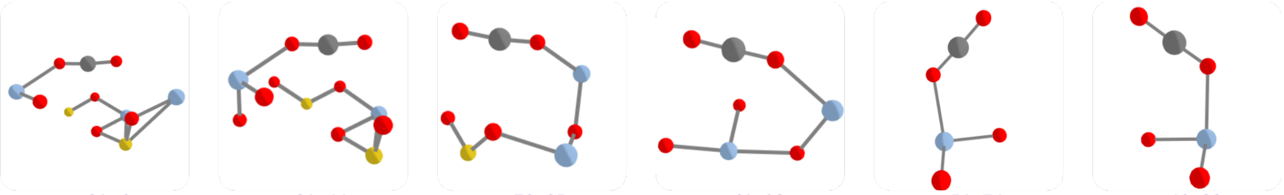

-81.6		-81.44		-79.25		-61.03		-51.74		-49.38	
2351	1.000	2358	1.000	2367	1.000	2382	1.000	2367	1.000	2371	1.000
1311	0.010	1315	0.014	1321	0.053	1329	0.036	1313	0.022	1316	0.010
587	0.091	590	0.090	604	0.035	608	0.041	621	0.058	624	0.067
564	0.085	565	0.085	546	0.112	594	0.050	612	0.048	606	0.046
189	0.006	194	0.006	181	0.007	170	0.005	199	0.002	212	0.004
143	0.000	140	0.000	138	0.001	97	0.000	88	0.000	91	0.000
108	0.001	113	0.001	85	0.001	66	0.000	68	0.001	78	0.000
60	0.002	73	0.001	59	0.003	55	0.001	26	0.001	34	0.002
55	0.003	65	0.002	36	0.001	8	0.002	18	0.001	34	0.002

Figure S4. Atomic representations of the 6 $\mathbf{CO_2}$ adsorption configurations displaying the lowest adsorption energies on the **(120)** surface of Mg_2SiO_4 in our calculations. Only atoms within a radius of 4 Å from the C center are displayed. Adsorption energies are denoted in purple, in kJ mol^{-1}. The arising spectroscopic features from these adsorptions are displayed under the adsorption energy, where the first column is the wavenumber in cm^{-1}, and the second column is the normalized intensity. Blue, dark yellow, red and gray balls represent Mg, Si, O and C atoms, respectively.

(101)

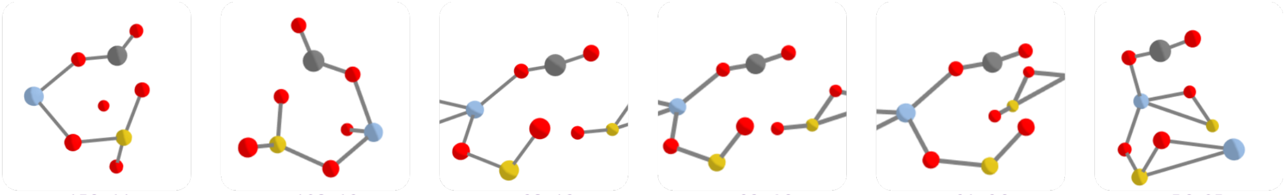

-158.11		-103.19		-93.12		-92.46		-91.96		-56.05	
1801	1.000	1821	1.000	2352	1.000	2359	1.000	2349	1.000	2321	1.000
1205	0.526	1209	0.517	1309	0.063	1315	0.064	1306	0.067	1284	0.038
764	0.335	741	0.350	564	0.159	575	0.121	562	0.162	584	0.118
720	0.042	729	0.026	550	0.148	548	0.156	542	0.165	572	0.286
457	0.005	441	0.002	193	0.005	197	0.005	189	0.005	225	0.007
331	0.013	324	0.034	151	0.001	154	0.001	162	0.002	136	0.002
244	0.021	220	0.019	125	0.001	113	0.001	138	0.001	120	0.003
139	0.008	135	0.010	63	0.005	72	0.009	79	0.006	63	0.004
64	0.007	35	0.004	54	0.006	52	0.003	63	0.006	41	0.006

Figure S5. Atomic representations of the 6 CO_2 adsorption configurations displaying the lowest adsorption energies on the **(101)** surface of Mg_2SiO_4 in our calculations. Only atoms within a radius of 4 Å from the C center are displayed. Adsorption energies are denoted in purple, in kJ mol^{-1}. The arising spectroscopic features from these adsorptions are displayed under the adsorption energy, where the first column is the wavenumber in cm^{-1}, and the second column is the normalized intensity. Blue, dark yellow, red and gray balls represent Mg, Si, O and C atoms, respectively.

(001)

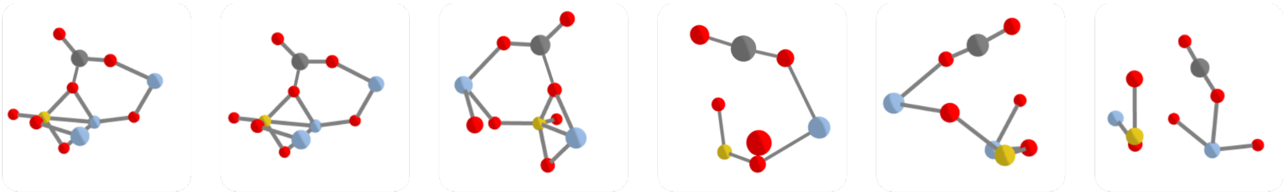

-182.72		-181.31		-117.83		-92.61		-67.16		-66.97	
1761	1.000	1763	1.000	1856	1.000	2359	1.000	2335	1.000	2361	1.000
1182	0.758	1193	0.782	1209	0.797	1304	0.031	1284	0.049	1312	0.004
788	0.193	791	0.233	735	0.088	611	0.157	595	0.071	614	0.074
753	0.022	749	0.025	715	0.146	565	0.137	546	0.280	582	0.086
459	0.006	460	0.004	435	0.027	225	0.005	217	0.002	176	0.006
367	0.019	369	0.013	314	0.121	123	0.001	128	0.004	134	0.001
276	0.019	263	0.019	219	0.025	114	0.001	93	0.002	89	0.001
67	0.016	74	0.020	120	0.020	46	0.003	52	0.006	63	0.002
48	0.001	59	0.001	40	0.003	29	0.004	39	0.006	22	0.001

Figure S6. Atomic representations of the 6 CO_2 adsorption configurations displaying the lowest adsorption energies on the **(001)** surface of Mg_2SiO_4 in our calculations. Only atoms within a radius of 4 Å from the C center are displayed. Adsorption energies are denoted in purple, in kJ mol^{-1}. The arising spectroscopic features from these adsorptions are displayed under the adsorption energy, where the first column is the wavenumber in cm^{-1}, and the second column is the normalized intensity. Blue, dark yellow, red and gray balls represent Mg, Si, O and C atoms, respectively.

(111)

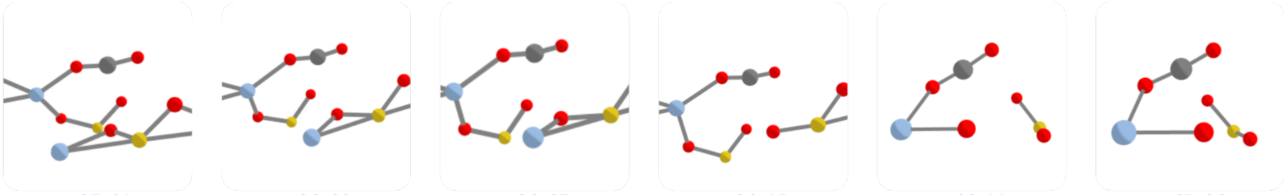

-87.01		-86.99		-86.87		-86.15		-66.11		-65.86	
2339	1.000	2334	1.000	2345	1.000	2351	1.000	2354	1.000	2351	1.000
1308	0.092	1306	0.081	1311	0.081	1314	0.053	1300	0.031	1298	0.030
576	0.104	578	0.107	572	0.120	567	0.127	608	0.082	602	0.093
527	0.228	523	0.239	538	0.184	550	0.172	593	0.117	583	0.136
188	0.007	194	0.007	194	0.008	187	0.006	214	0.003	212	0.002
175	0.003	184	0.005	168	0.002	164	0.003	110	0.002	112	0.002
123	0.005	130	0.000	127	0.002	125	0.000	97	0.001	101	0.001
68	0.009	82	0.031	74	0.004	76	0.009	55	0.002	62	0.004
61	0.016	71	0.007	50	0.017	50	0.006	39	0.003	38	0.002

Figure S7. Atomic representations of the 6 CO_2 adsorption configurations displaying the lowest adsorption energies on the **(111)** surface of Mg_2SiO_4 in our calculations. Only atoms within a radius of 4 Å from the C center are displayed. Adsorption energies are denoted in purple, in kJ mol^{-1}. The arising spectroscopic features from these adsorptions are displayed under the adsorption energy, where the first column is the wavenumber in cm^{-1}, and the second column is the normalized intensity. Blue, dark yellow, red and gray balls represent Mg, Si, O and C atoms, respectively.

(110)

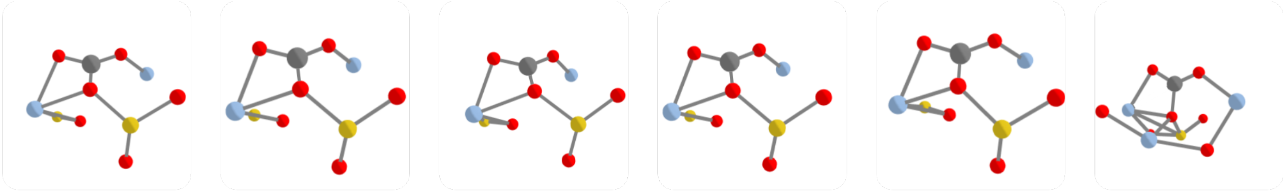

-209.20		-209.14		-209.12		-208.38		-208.14		-173.42	
1597	1.000	1596	1.000	1599	1.000	1594	1.000	1598	1.000	1648	1.000
1256	0.628	1256	0.622	1257	0.702	1253	0.880	1256	0.905	1253	0.736
816	0.081	816	0.083	815	0.087	816	0.058	816	0.055	813	0.077
741	0.059	741	0.059	741	0.058	741	0.070	741	0.070	760	0.019
512	0.016	512	0.015	512	0.047	512	0.109	512	0.094	516	0.008
398	0.029	398	0.029	398	0.090	398	0.129	398	0.136	407	0.026
269	0.017	269	0.017	269	0.023	270	0.038	271	0.039	230	0.050
206	0.019	206	0.019	208	0.015	207	0.011	207	0.012	166	0.006
173	0.022	173	0.022	173	0.024	170	0.026	176	0.026	115	0.013

Figure S8. Atomic representations of the 6 CO_2 adsorption configurations displaying the lowest adsorption energies on the **(110)** surface of Mg_2SiO_4 in our calculations. Only atoms within a radius of 4 Å from the C center are displayed. Adsorption energies are denoted in purple, in kJ mol^{-1}. The arising spectroscopic features from these adsorptions are displayed under the adsorption energy, where the first column is the wavenumber in cm^{-1}, and the second column is the normalized intensity. Blue, dark yellow, red and gray balls represent Mg, Si, O and C atoms, respectively.

(021)

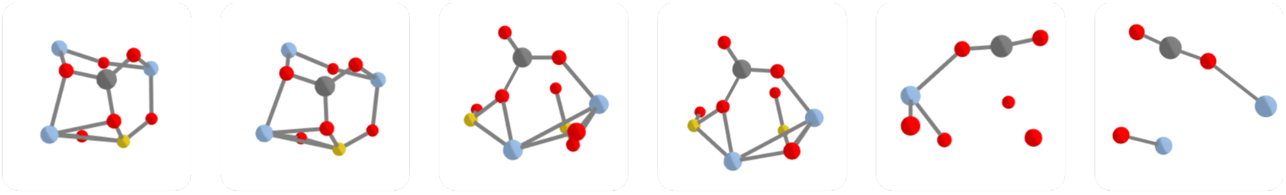

-232.23		-231.85		-133.86		-133.63		-86.93		-81.56	
**1640**	1.000	**1639**	1.000	**1770**	1.000	**1771**	1.000	**2360**	1.000	**2375**	1.000
**1205**	0.835	**1203**	0.841	**1200**	0.706	**1200**	0.692	**1311**	0.058	**1322**	0.076
**832**	0.081	**833**	0.084	**766**	0.284	**765**	0.393	**584**	0.083	**595**	0.032
**778**	0.046	**778**	0.048	**723**	0.063	**725**	0.065	**563**	0.151	**540**	0.180
**520**	0.003	**521**	0.003	**454**	0.002	**453**	0.026	**202**	0.005	**175**	0.004
**426**	0.007	**427**	0.008	**347**	0.024	**345**	0.031	**146**	0.001	**115**	0.001
**283**	0.028	**280**	0.047	**225**	0.016	**224**	0.029	**117**	0.001	**80**	0.001
**247**	0.032	**242**	0.033	**184**	0.013	**183**	0.014	**70**	0.001	**46**	0.004
**172**	0.028	**172**	0.028	**77**	0.012	**75**	0.014	**52**	0.004	**11**	0.002

Figure S9. Atomic representations of the 6 $\mathbf{CO_2}$ adsorption configurations displaying the lowest adsorption energies on the **(021)** surface of Mg_2SiO_4 in our calculations. Only atoms within a radius of 4 Å from the C center are displayed. Adsorption energies are denoted in purple, in kJ mol^{-1}. The arising spectroscopic features from these adsorptions are displayed under the adsorption energy, where the first column is the wavenumber in cm^{-1}, and the second column is the normalized intensity. Blue, dark yellow, red and gray balls represent Mg, Si, O and C atoms, respectively.

(010)

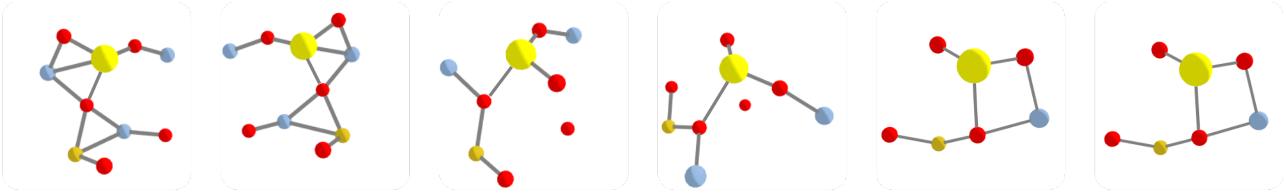

-171.33		-170.02		-113.72		-112.67		-106.36		-106.33	
**1080**	1.000	**1078**	1.000	**1216**	0.882	**1218**	0.954	**1181**	1.000	**1181**	1.000
**989**	0.484	**985**	0.438	**1044**	1.000	**1051**	1.000	**980**	0.874	**980**	0.872
**538**	0.080	**536**	0.078	**497**	0.103	**494**	0.103	**510**	0.060	**510**	0.060
**457**	0.202	**455**	0.188	**317**	0.228	**320**	0.243	**445**	0.353	**446**	0.353
**321**	0.021	**321**	0.022	**207**	0.024	**201**	0.022	**276**	0.087	**276**	0.087
**209**	0.041	**209**	0.034	**169**	0.003	**174**	0.007	**168**	0.012	**168**	0.012
**167**	0.015	**166**	0.014	**107**	0.015	**110**	0.013	**103**	0.013	**103**	0.013
**140**	0.069	**137**	0.086	**81**	0.011	**76**	0.012	**80**	0.011	**80**	0.011
**114**	0.025	**118**	0.018	**61**	0.002	**42**	0.007	**45**	0.006	**46**	0.006

Figure S10. Atomic representations of the 6 $\mathbf{SO_2}$ adsorption configurations displaying the lowest adsorption energies on the **(010)** surface of Mg_2SiO_4 in our calculations. Only atoms within a radius of 4 Å from the S center are displayed. Adsorption energies are denoted in purple, in kJ mol^{-1}. The arising spectroscopic features from these adsorptions are displayed under the adsorption energy, where the first column is the wavenumber in cm^{-1}, and the second column is the normalized intensity. Blue, dark yellow, red and light yellow balls represent Mg, Si, O and S atoms, respectively.

(120)

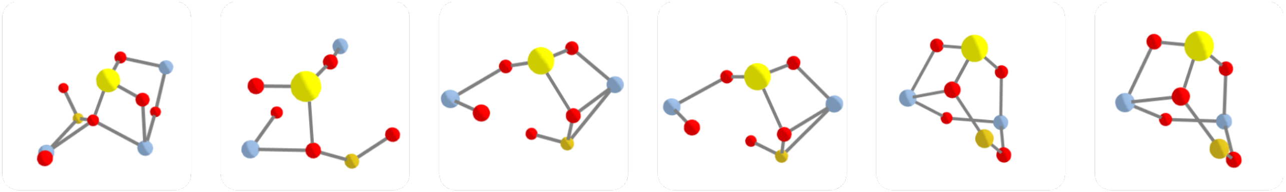

-162.64		-161.04		-149.10		-148.86		-137.96		-137.91	
1161	1.000	1187	1.000	1138	1.000	1140	1.000	1139	1.000	1138	1.000
1089	0.881	1104	0.493	1031	0.547	1033	0.556	1062	0.405	1060	1.000
599	0.153	527	0.034	578	0.079	576	0.079	582	0.249	582	0.217
461	0.319	417	0.158	479	0.180	473	0.177	463	0.207	463	0.212
280	0.036	258	0.005	336	0.063	335	0.064	364	0.028	364	0.031
264	0.009	209	0.005	209	0.018	207	0.017	236	0.038	233	0.037
172	0.046	154	0.017	174	0.015	172	0.014	221	0.003	220	0.003
139	0.011	129	0.009	147	0.011	156	0.011	165	0.032	165	0.030
89	0.012	107	0.021	86	0.015	89	0.011	120	0.028	118	0.027

Figure S11. Atomic representations of the 6 $\mathbf{SO_2}$ adsorption configurations displaying the lowest adsorption energies on the **(120)** surface of Mg_2SiO_4 in our calculations. Only atoms within a radius of 4 Å from the S center are displayed. Adsorption energies are denoted in purple, in kJ mol^{-1}. The arising spectroscopic features from these adsorptions are displayed under the adsorption energy, where the first column is the wavenumber in cm^{-1}, and the second column is the normalized intensity. Blue, dark yellow, red and light yellow balls represent Mg, Si, O and S atoms, respectively.

(101)

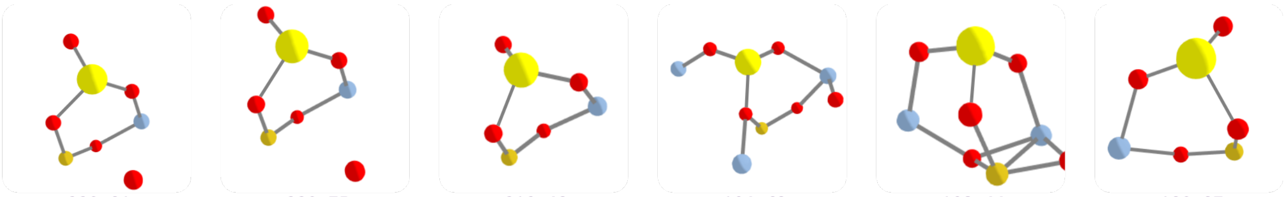

-220.81		-220.75		-219.48		-194.68		-193.44		-180.87	
1256	1.000	1233	0.780	1201	0.769	1153	1.000	1096	1.000	1200	1.000
1011	0.977	1020	1.000	1008	1.000	1081	0.291	1043	0.510	1021	0.980
552	0.080	559	0.231	581	0.268	598	0.048	591	0.163	565	0.203
469	0.411	470	0.480	470	0.402	512	0.183	477	0.309	500	0.474
305	0.018	336	0.028	354	0.018	351	0.022	344	0.003	324	0.025
266	0.012	271	0.023	281	0.007	255	0.005	278	0.013	234	0.030
157	0.019	169	0.011	182	0.013	233	0.019	231	0.011	151	0.009
100	0.013	85	0.013	72	0.021	157	0.007	156	0.009	92	0.027
66	0.012	21	0.001	35	0.004	132	0.018	126	0.020	34	0.001

Figure S12. Atomic representations of the 6 $\mathbf{SO_2}$ adsorption configurations displaying the lowest adsorption energies on the **(101)** surface of Mg_2SiO_4 in our calculations. Only atoms within a radius of 4 Å from the S center are displayed. Adsorption energies are denoted in purple, in kJ mol^{-1}. The arising spectroscopic features from these adsorptions are displayed under the adsorption energy, where the first column is the wavenumber in cm^{-1}, and the second column is the normalized intensity. Blue, dark yellow, red and light yellow balls represent Mg, Si, O and S atoms, respectively.

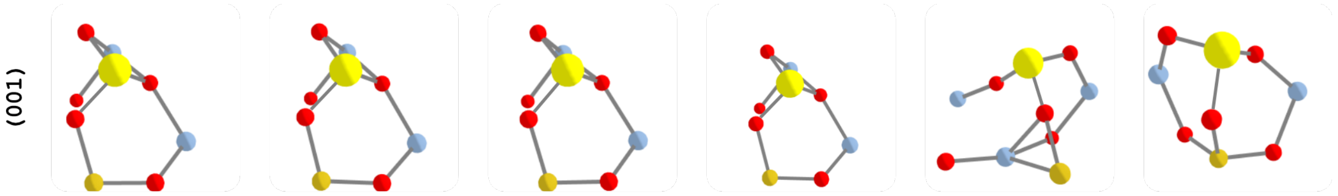

-339.30		-339.29		-339.28		-339.25		-321.28		-295.52	
1058	1.000	1058	1.000	1057	1.000	1058	1.000	1030	0.665	1034	0.324
923	0.984	923	0.987	924	0.961	922	0.980	980	1.000	1012	1.000
665	0.173	665	0.176	665	0.173	665	0.169	608	0.259	628	0.564
566	0.559	566	0.566	566	0.554	566	0.550	505	0.180	536	0.057
338	0.010	339	0.011	339	0.011	338	0.010	362	0.012	368	0.196
312	0.005	312	0.004	312	0.004	312	0.004	319	0.030	332	0.010
281	0.008	282	0.008	282	0.008	281	0.008	280	0.002	280	0.041
220	0.074	220	0.077	220	0.079	220	0.081	204	0.043	191	0.095
138	0.013	138	0.012	138	0.012	138	0.012	148	0.035	85	0.011

Figure S13. Atomic representations of the 6 SO_2 adsorption configurations displaying the lowest adsorption energies on the **(001)** surface of Mg_2SiO_4 in our calculations. Only atoms within a radius of 4 Å from the S center are displayed. Adsorption energies are denoted in purple, in kJ mol^{-1}. The arising spectroscopic features from these adsorptions are displayed under the adsorption energy, where the first column is the wavenumber in cm^{-1}, and the second column is the normalized intensity. Blue, dark yellow, red and light yellow balls represent Mg, Si, O and S atoms, respectively.

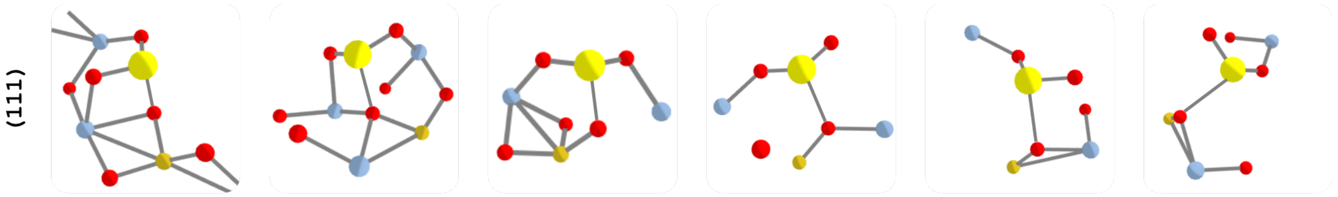

-189.29		-164.06		-140.01		-139.00		-130.43		-123.85	
1105	1.000	1169	0.903	1122	1.000	1271	1.000	1245	0.828	1300	0.621
1064	0.535	1078	1.000	992	0.611	1106	0.697	1062	1.000	1081	1.000
603	0.137	576	0.134	577	0.155	553	0.155	555	0.094	545	0.146
504	0.272	456	0.361	520	0.186	318	0.186	342	0.232	310	0.129
341	0.017	328	0.004	384	0.047	214	0.047	204	0.012	219	0.013
330	0.024	245	0.030	271	0.012	197	0.012	160	0.029	161	0.002
236	0.030	179	0.043	208	0.015	126	0.015	118	0.017	109	0.013
168	0.010	156	0.022	157	0.015	93	0.044	89	0.006	76	0.005
122	0.014	147	0.008	116	0.030	66	0.006	70	0.005	48	0.000

Figure S14. Atomic representations of the 6 SO_2 adsorption configurations displaying the lowest adsorption energies on the **(111)** surface of Mg_2SiO_4 in our calculations. Only atoms within a radius of 4 Å from the S center are displayed. Adsorption energies are denoted in purple, in kJ mol^{-1}. The arising spectroscopic features from these adsorptions are displayed under the adsorption energy, where the first column is the wavenumber in cm^{-1}, and the second column is the normalized intensity. Blue, dark yellow, red and light yellow balls represent Mg, Si, O and S atoms, respectively.

(110)

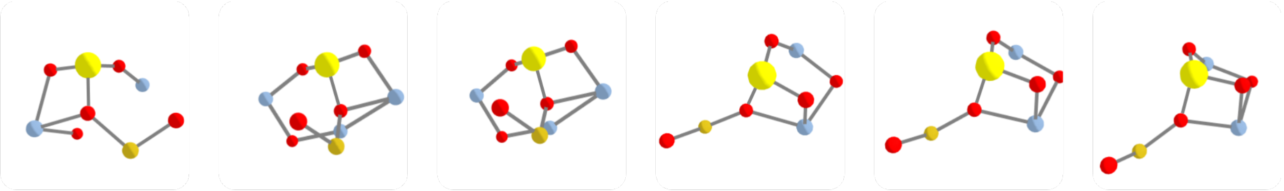

-268.62		-258.59		-258.58		-238.68		-238.36		-238.27	
**1097**	1.000	**1036**	1.000	**1034**	1.000	**1075**	1.000	**1071**	1.000	**1073**	1.000
**1008**	0.673	**1002**	0.577	**1001**	0.444	**1021**	0.830	**1018**	0.844	**1017**	0.880
**571**	0.203	**604**	0.263	**603**	0.223	**604**	0.267	**603**	0.267	**604**	0.318
**484**	0.118	**526**	0.096	**525**	0.112	**489**	0.298	**489**	0.298	**488**	0.291
**349**	0.015	**406**	0.017	**405**	0.017	**356**	0.019	**356**	0.019	**357**	0.016
**260**	0.007	**303**	0.020	**300**	0.019	**297**	0.044	**301**	0.044	**300**	0.045
**228**	0.015	**224**	0.047	**224**	0.028	**254**	0.003	**257**	0.003	**255**	0.002
**164**	0.019	**165**	0.017	**164**	0.022	**177**	0.049	**175**	0.049	**174**	0.041
**149**	0.042	**116**	0.009	**117**	0.011	**111**	0.041	**108**	0.041	**108**	0.036

Figure S15. Atomic representations of the 6 $\mathbf{SO_2}$ adsorption configurations displaying the lowest adsorption energies on the **(110)** surface of Mg_2SiO_4 in our calculations. Only atoms within a radius of 4 Å from the S center are displayed. Adsorption energies are denoted in purple, in kJ mol^{-1}. The arising spectroscopic features from these adsorptions are displayed under the adsorption energy, where the first column is the wavenumber in cm^{-1}, and the second column is the normalized intensity. Blue, dark yellow, red and light yellow balls represent Mg, Si, O and S atoms, respectively.

(021)

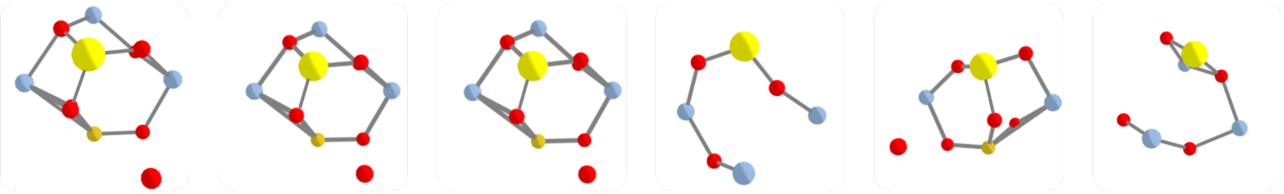

-316.10		-316.08		-315.91		-294.51		-270.94		-226.57	
**1153**	0.747	**1153**	0.747	**1147**	0.766	**1040**	0.404	**1075**	1.000	**1039**	0.285
**828**	1.000	**829**	1.000	**825**	1.000	**940**	1.000	**1022**	0.484	**947**	1.000
**605**	0.282	**605**	0.282	**608**	0.282	**491**	0.048	**596**	0.213	**576**	0.044
**537**	0.185	**537**	0.186	**543**	0.184	**309**	0.014	**530**	0.166	**307**	0.009
**363**	0.023	**363**	0.023	**367**	0.026	**254**	0.030	**411**	0.031	**222**	0.082
**295**	0.005	**295**	0.005	**293**	0.003	**136**	0.008	**281**	0.011	**190**	0.026
**273**	0.026	**274**	0.025	**277**	0.027	**127**	0.028	**238**	0.017	**141**	0.049
**237**	0.027	**236**	0.028	**232**	0.028	**96**	0.002	**160**	0.017	**114**	0.069
**157**	0.024	**157**	0.024	**153**	0.024	**39**	0.015	**138**	0.012	**85**	0.006

Figure S16. Atomic representations of the 6 $\mathbf{SO_2}$ adsorption configurations displaying the lowest adsorption energies on the **(021)** surface of Mg_2SiO_4 in our calculations. Only atoms within a radius of 4 Å from the S center are displayed. Adsorption energies are denoted in purple, in kJ mol^{-1}. The arising spectroscopic features from these adsorptions are displayed under the adsorption energy, where the first column is the wavenumber in cm^{-1}, and the second column is the normalized intensity. Blue, dark yellow, red and light yellow balls represent Mg, Si, O and S atoms, respectively.
